# Enhancing the Performance of PLA Nonwoven Fabrics Through Plasma Treatments for Superior Active-Molecule Retention

**DOI:** 10.3390/polym17111482

**Published:** 2025-05-27

**Authors:** Norma Mallegni, Serena Coiai, Francesca Cicogna, Luca Panariello, Caterina Cristallini, Stefano Caporali, Elisa Passaglia

**Affiliations:** 1 CNR-Consiglio Nazionale delle Ricerche Istituto di Chimica dei Composti OrganoMetallici (CNR-ICCOM), SS di Pisa, Area della Ricerca di Pisa, 56124 Pisa, Italy; norma.mallegni@pi.iccom.cnr.it (N.M.); serena.coiai@pi.iccom.cnr.it (S.C.); francesca.cicogna@pi.iccom.cnr.it (F.C.); 2 Dipartimento di Ingegneria Civile e Industriale, DICI, Università di Pisa, Largo L. Lazzarino 1, 56122 Pisa, Italy; luca.panariello@ing.unipi.it; 3 CNR-Consiglio Nazionale delle Ricerche Istituto per i Processi Chimico-Fisici (CNR-IPCF), SS di Pisa, Largo L. Lazzarino 1, 56122 Pisa, Italy; caterina.cristallini@cnr.it; 4 Dipartimento di Ingegneria Industriale, DIEF, Università di Firenze, Via S. Marta 3, 50139 Firenze, Italy; stefano.caporali@unifi.it

**Keywords:** cold plasma, EGCG, green tea extract, NWF, PLA, surface functionalization

## Abstract

Polylactic acid (PLA) is a promising biobased polymer celebrated for its biocompatibility, biodegradability, and advantageous mechanical properties. However, its inherent hydrophobicity and lack of hydrophilic functional groups restrict its application in advanced uses, such as nonwoven fabrics (NWFs) for masks, diapers, and biomedical products. This study explores the application of cold plasma treatments to modify the surface of PLA-based NWFs using oxygen and oxygen–argon gas mixtures. We varied power levels and exposure times to optimize surface activation. The samples treated with plasma under different conditions were analyzed to understand the impact of these treatments on the surface functionalization, morphology, and thermal properties of PLA_NWF. Additionally, as a proof of concept, the plasma-treated samples were dip-coated in green tea extract, which is rich in (-)-epigallocatechin gallate (EGCG), a natural antioxidant. The findings demonstrate that plasma treatment significantly enhances the adhesion and functionality of the active ingredient, thereby paving the way for innovative sustainable applications of surface-activated PLA-NWFs in the biomedical and cosmetic sectors or food preservation.

## 1. Introduction

Nowadays, there is a pressing need to substitute non-biodegradable substances derived from petroleum with biodegradable options obtained from renewable resources, especially for single-use items, like sanitaryware. In this regard, polylactic acid (PLA) is recognized as a highly promising bio-based polymer due to its biocompatibility with living organisms, biodegradability under industrial composting conditions, recyclability, and excellent mechanical characteristics [1]. PLA is produced from renewable resources, such as corn, potatoes, and sugarcane [2], and it has several advantageous properties, such as high mechanical strength, non-toxicity, and non-irritation, while also being easy to process. It exhibits performance characteristics similar to polyethylene terephthalate (PET) and polypropylene (PP) [1,3]. PLA finds extensive use in disposable consumer goods, particularly for packaging, such as loose-fill packaging, compost bags, food containers, and disposable tableware [4]. It is also widely used in the textile industry and, together with its copolymer derivatives, it has important biomedical applications, including surgical sutures, implants [5], scaffolds for bone regeneration [6], and carriers for drug delivery [7]. Owing to its biodegradability, biocompatibility, and non-toxicity, PLA is also gaining significant attention in the nonwoven industry to obtain biodegradable nonwoven fabrics (PLA-NWFs) for possible uses as masks, diapers, civil wipes, wet facial towels, beauty products, and sanitary pads, alternatives to those made by oil-based polymers [7,8,9]. However, PLA is relatively hydrophobic and suffers from the lack of functional hydrophilic groups, partially inhibiting the possible uses in the above-mentioned applications. Indeed, PLA is characterized by a water contact angle ranging from 70 to 80 degrees [10]. Many surface modification strategies have been developed to enhance the wettability of PLA films and fabrics through chemical and physical activation [10,11,12,13]. Among these methods, physical modification—particularly using plasma treatments—has proven versatile. Especially cold plasma stands out as a safe and cost-effective technology that significantly modifies the surface characteristics of polymer-based films and textiles, particularly nonwoven fabrics (NWFs), without negatively impacting their bulk properties [14,15,16]. Despite the promising results of plasma treatments, the precise relationship between plasma parameters (such as gas composition, power, and exposure time) and the extent of surface modification remains unclear, particularly for PLA-NWF. This gap limits the ability to fine-tune surface properties for specific applications. In biomedical applications, plasma has been widely used to immobilize active and functional derivatives, including cyclodextrins, dopamine, and antibacterial agents, on the surface layers of various substrates [17,18,19,20,21], allowing for appropriate and tunable modifications while preserving the shape and bulk properties of the original materials and goods.

The surface activation of PLA-based films and textiles using plasma typically involves inert or oxidizing gases, as well as functionalizing agents introduced directly into the plasma chamber. This process results in both physical and chemical modifications [11]. The research highlights notable outcomes, including increased surface roughness [22,23], improved wettability and hydrophilicity [6,24,25,26], enhanced adhesion capability [27], the introduction of novel functionalities [28,29,30], and the grafting of specific chemicals [31,32]. These surface modifications, often followed by the immobilization of active ingredients, are widely explored for applications in packaging [23], biomedicine, and cosmetics [6,22,24,32,33]. Depending on the type of gas and experimental conditions, different mechanisms of surface structure alteration are discussed, involving mechanisms such as bond breaking with and without the release of small molecules, crosslinking, and insertion of oxygenated functionalities through radical reactions [11,29]. In general, gases such as N_2_, Ar, He, C_6_F_14_, SF_6_, or very low-pressure air atmospheres generate unstable intermediates, mainly radicals. These radicals can quickly react with oxygen once the samples are exposed to air, along with active chemicals introduced into the plasma chamber [30,34] during or after plasma activation [31,35]. Such processes are used for fixing nanoparticles [22,36] or for simply modifying the wettability of the surfaces [32,37]. On the other hand, oxidizing gases (such as O_2_, air) or specific reactive atmospheres are employed to directly functionalize the PLA polymer films or fabric surfaces [23,24,28,38]. These modifications are aimed at enhancing packaging applications [39] or improving drug anchoring (to facilitate the controlled release) [38], and cell adhesion [40] in biomedical applications.

Few studies have investigated the relationships between the extent of surface modifications in PLA substrates and the plasma parameters used for their activation. Some data are available on PLA films, specifically concerning plasma exposure times [6,23,24,26,31,34]. However, for PLA-based textiles, the information remains limited on the exposure time and power settings for cold plasma treatments using air and C_6_F_14_ [32], as well as post-treatment with acidic or alkaline solutions [30], or in the presence of reactive acrylic acid [41]. These studies aim to optimize wettability, liquid absorption [32], mechanical properties [30], and hydrophilicity for bone tissue engineering applications [41].

In this study, we present a comprehensive investigation of the cold plasma activation of PLA-NWFs. We varied the power of the plasma generator and the exposure time, utilizing both oxygen and a mixture of oxygen and argon. These gases were selected based on their known ability to introduce oxygen-containing functional groups, which enhance wettability and adhesion properties. The plasma-treated samples were analyzed for their functional, morphological, and thermal characteristics to determine the relationship between the experimental conditions and the degree of surface modification caused by different treatments. To explore the potential of plasma-treated PLA-NWFs for biomedical and antibacterial applications, we immobilized a green tea extract rich in (-)-epigallocatechin gallate (EGCG), a natural polyphenol known for its antioxidant and antibacterial properties [42,43]. The rationale behind choosing EGCG lies in its potential to enhance the bioactivity of PLA-NWFs for applications such as wound dressings, antimicrobial textiles, or packaging with extended shelf-life properties. We thoroughly examined the extract’s absorption and the EGCG retention capability in both pristine and plasma-treated PLA-NWF samples, along with the morphological and thermal characteristics of the final products.

This study aims to optimize plasma treatment parameters for PLA-NWF modification and evaluate its potential in functional bioactive applications. The results could contribute to the development of advanced biodegradable textiles with enhanced wettability and biofunctional properties.

## 2. Materials and Methods

### 2.1. Materials

Unless otherwise specified, all the materials employed in this study were used as received without further purification. PLA nonwoven fabric (PLA-NWF, diameter: 20–25 μm) was purchased from Weifang Shuntai Textile Co., Ltd., Qingzhou, Weifang City, Shandong province, China. Green tea bags were purchased from a local market (packaged in Italy). Chloroform ACS reagent ≥ 99.8%, chloroform HPLC grade ≥ 99.8% (ethanol-stabilized), ethanol HPLC grade ≥ 99.8%, 2,2-diphenyl-1-picrylhydrazyl hydrate (DPPH), hexamethyl pararosaniline chloride (crystal violet, CV), and (-)-epigallocatechin gallate (EGCG) were purchased from Sigma-Aldrich (St. Louis, MO, USA); methanol 99+% extra pure was purchased from Acros Organics (Geel, Belgium). Deionized ultrapure water (resistivity: 18.2 MOhm cm) was obtained using a Milli-Q system (Millipore, Bedford, MA, USA).

### 2.2. Green Tea Extraction and Characterization of Extracts

The green tea extract (GTex) was obtained by extracting 58 g of green tea powder in a 450 mL of ethanol/water 3/1 (*v*/*v*) mixture under magnetic stirring for 1 week at room temperature. After filtration with a paper filter, GTex was stored in a black glass bottle at room temperature. The content of epigallocatechin gallate (EGCG) in the extract was determined using UV-Vis spectroscopy. More specifically, by using the absorbance at 273 nm (more intense absorption band of EGCG) and the molar extinction coefficient of EGCG at that wavelength, it is possible to evaluate the concentration of EGCG in the extract. The molar extinction coefficient of EGCG at 273 nm was determined by a calibration curve (Figure S1) obtained by measuring the absorbance at 273 nm of solutions at different concentrations of EGCG (HPLC standard) in an ethanol/water 3/1 (*v*/*v*) mixture. A linear fitting of the absorbance at 273 nm, as a function of the molar concentration of EGCG solutions, allowed the determination of the molar extinction coefficient of EGCG at 273 nm: ε~9300 M^−1^ cm^−1^ (R^2^ value of fitting = 0.99982). To evaluate the concentration of GTex, 10 μL of GTex was diluted with 5 mL of an ethanol/water 3/1 (*v*/*v*) mixture, and the absorbance of this solution was determined. Using the molar extinction coefficient of ECGC at 273 nm, we obtained a concentration of EGCG in GTex of 2.8 × 10^−2^ M.

The radical scavenging activity of the EGCG (HPLC standard) solution and GTex was evaluated using the 2,2-diphenyl-1-picrylhydrazyl (DPPH) assay method. In the test, we used a diluted solution of GTex with a concentration in EGCG of 2.8 × 10^−4^ M and prepared a solution of EGCG (HPLC standard) in ethanol (1.6 × 10^−4^ M) and a DPPH solution in methanol (7.6 × 10^−5^ M). A total of 3 mL of the DPPH solution was mixed with different aliquots of EGCG solution or GTex to obtain final concentrations of ECGC ranging from 9.3 × 10^−6^ M to 4.6 × 10^−7^ M, and from 3.2 × 10^−6^ M and 1.6 × 10^−7^ M in the case of the ECGC (HPLC standard) solution. MeOH was added to each solution to reach a final volume of 3.1 mL. Additionally, a blank DPPH solution was prepared by adding 0.1 mL of MeOH to the DPPH solution. The percentage of DPPH reduction (*I*%) (Equation (1)) was calculated as a function of the antioxidant concentration, and linear fitting of the experimental data was performed. The EC_50_ value was determined as the antioxidant concentration corresponding to I% = 50%. (EC_50_ of GTex = 5.4 μM and of EGCG = 2.07 μM)

(1) $$I\;\%=\frac{\left(A_{0}-A_{t}\right)}{A_{0}}\times 100$$

where:

*A*_0_ is the absorbance of the DPPH solution in the absence of the antioxidant after 24 h. *A_t_* is the absorbance of the DPPH solution in the presence of the antioxidant at the end of the reaction.

### 2.3. PLA-NWF Modification

Plasma activation: a continuous plasma reactor (Tucano, Gambetti, Italy) with a working area of 118 mm × 310 mm and a chamber capacity of approximately 5.5 L was used to treat PLA-NWF pieces measuring 9 × 4 cm. The treatment procedure consisted of four steps: (i) reducing the pressure in the reaction chamber from ambient pressure to about 0.2 mbar; (ii) stabilizing the internal pressure for five seconds; (iii) introducing selected gas (oxygen or oxygen–argon flux as reported in Table S1), and (iv) plasma reaction. The total pressure varied from 0.3 to 0.8 mbar depending on the gas flux during the plasma treatment; after the planned exposure time, venting to ambient pressure took about 150 s. Each sample was treated on both sides. The PLA-NWF samples (about 9 × 4 cm) were treated by systematically changing the plasma conditions as reported in Table S1. In summary, the plasma power was varied between 50 and 100 W, and the exposure time ranged from 30 to 120 s. The gas flow rate was fixed at 15 sccm for each gas. For the treatments conducted using both O_2_ and Ar, the flow ratio between the two gases was maintained at 1:1.

Treatment of PLA-NWF and plasma-treated PLA-NWF with crystal violet (CV): a sodium acetate/acetic acid buffer (freshly prepared, pH = 4.7) was used to solubilize hexamethyl pararosaniline chloride (crystal violet, CV). A total of 1.8 mg of CV was dissolved in 100 mL of the buffer to a final concentration of 4.4 × 10^−5^ M. Small pieces of plasma-treated PLA-NWF, and PLA-NWF (blank) (2–3 mg each), were soaked in 1 mL of CV solution and 1 mL of water for 24 h. Subsequently, each piece was washed in water, dried under vacuum, and dissolved in chloroform. The resulting solutions were analyzed by UV-vis spectroscopy to determine the amount of absorbed CV. Specifically, the solution’s absorbance at 590 nm (the absorption maximum of CV) was measured and normalized to the weight units of each sample.

### 2.4. Coating of PLA-NWF and Plasma-Treated PLA-NWF with Green Tea Extract (GTex)

PLA-NWF and plasma-treated PLA-NWF, approximately 4 × 4 cm in size, were dipped in 5 mL of GTex with magnetic stirring for 5 min. Then, they were removed from GTex and left to dry at room temperature overnight and dried in a vacuum oven at 30 °C for about 1 h. Samples PLA_NWF_GTex, PLA_NWF_100_60_O_2__GTex, and PLA_NWF_100_60_O_2__Ar_GTex were obtained, corresponding to PLA_NWF, PLA_NWF_100_60_O_2_, and PLA_NWF_100_60_O_2__Ar (see Table S1) dip-coated with GTex.

### 2.5. Samples’ Characterization

The infrared spectra on PLA-NWF samples were acquired with the Perkin-Elmer Spectrum Two FTIR Spectrophotometer (Norwalk, CT, USA), equipped with a diamond-crystal attenuated total reflectance (ATR) accessory. Each ATR-FTIR spectrum was obtained by applying 32 scans at a resolution of 4 cm^−1^. The water-extracted fraction was evaporated to dryness, then resuspended with a few drops of chloroform and deposited onto a KBr disk. The ethanol-extracted fraction, on the other hand, was directly applied to the KBr disk.

The UV-vis absorption spectra were recorded at room temperature using a Jasco V750 spectrophotometer (Jasco International Co., Ltd., Tokyo, Japan), acquiring data in the 700–200 nm range with 10 scans at a resolution of 1 nm.

Size exclusion chromatography (SEC) analysis was carried out with the Agilent Technologies 1260 Series instrument (Agilent Technologies, Santa Clara, CA, USA) equipped with a degasser, an isocratic HPLC pump, a refractive index (RI) detector, and one pre-column PLgel 5 μm and two PLgel MiniMIX-D 5 μm columns conditioned at 35 ◦C. Measurements of the starting and modified samples were carried out in CHCl_3_ at a flow rate of 0.3 mL min^−1^. Polystyrene standards in the range of 500 to 3 × 10^5^ g mol^−1^ number-average molecular weight were used to calibrate the system. A total of 1–2 mg of polymer samples was dissolved in 1 mL of CHCl_3_ and filtered through a 0.20 μm syringe filter before analysis. Agilent ChemStation software was utilized to compute the number-average and weight-average molecular weights.

TGA analysis of the samples was carried out using a Seiko EXSTAR 7200 TGA/DTA instrument (Masterlab, Milan, Italy). Each measurement utilized 5–10 mg of sample and was performed in triplicate under air or nitrogen flow of 200 mL min^−1^. The temperature range was 30–700 °C with a heating rate of 10 °C min^−1^.

Differential Scanning Calorimetry (DSC) analyses were performed with the Perkin-Elmer DSC-4000 differential scanning calorimeter thermal analyzer (Perkin-Elmer, Waltham, MA, USA), cooled with water. Before analysis, the instrument was calibrated using zinc (melting point: 419.5 °C) and indium (melting point: 156.6 °C, ∆H = 28.5 Jg^−1^). The samples (5–10 mg) were subjected to the following thermal cycle: first heating at a rate of 10 °C min^−1^ from 30 °C to 200 °C keeping the temperature at 200 °C for 3 min; cooling from 200 to 30 °C at 10 °C min^−1^; keeping a low temperature at 30 °C for 3 min; and second heating from 30 to 200 °C at 10 °C min^−1^. Crystallization and melting temperatures were evaluated from the first and second heating, and the relative enthalpies were calculated from the integrated areas of crystallization and melting peaks, using the instrument software Pyris V9.0.

Scanning electron microscopy (SEM) analyses were conducted at the “Centro per l’Integrazione della Strumentazione Scientifica, Università di Pisa (CISUP)” using an FEI Quanta 450 FEG-SEM by Thermofischer Scientific (Pardubice, Czech Republic), and equipped with an EDX spectrometer Bruker QUANTAX XFlash Detector 6|10, Berlin, Germany. For enhanced resolution, all samples underwent platinum sputter-coating.

X-ray photoelectron spectroscopy (XPS) was conducted in an ultrahigh vacuum (UHV, 10^−9^ mbar) system equipped with a VSW HAC 500 hemispherical electron-energy analyzer (model HA100, VSW Scientific Instrument, Ltd., Manchester, UK) and a non-monochromatic Mg Kα X-ray source (1.254 keV, model TA10) operating at 120 W (12 kV × 10 mA). Samples were analyzed as received, fixed to the sample holder with conductive carbon tape, introduced into the UHV under an inert nitrogen (N_2_) flux, and kept in the introduction chamber for 24 h before measurement. Survey and high-resolution spectra were acquired in the constant analyzer energy (CAE) mode at a pass energy (E_pas_) of 22 eV, with step sizes of 1.0 eV and 0.1 eV, respectively. Spectral peaks were fitted using CasaXPS software with Gauss–Lorentz curves after subtracting a Shirley-type background. The binding energy (BE) scale was calibrated using the aliphatic C1s component at 284.8 ± 0.1 eV as a reference.

### 2.6. Migration Tests

Migration tests were conducted in triplicate on PLA_NWF_GTex, PLA_NWF_100_60_O_2__GTex, and PLA_NWF_100_60_O_2__Ar_GTex. Approximately 4.5 mg of each sample was immersed in 5 mL of deionized water at room temperature and stirred magnetically at 250 rpm for up to 1440 min (24 h). The release kinetics of EGCG were monitored using UV-vis spectroscopy, with spectra recorded at regular intervals. Two mathematical models were applied to model the release kinetics: the Korsmeyer–Peppas (power-law) Equation (2) and the Elovich Equation (3).

(2) $$\frac{M_{t}}{M_{\infty }}=k_{KP}t^{n}$$

(3) $$Q_{t}=\left(\frac{1}{\beta }\right)ln\left(\alpha \beta \right)+\left(\frac{1}{\beta }\right)ln\left(t\right)$$

where *M_t_*/*M_∞_* represents the fraction of EGCG released at time *t* relative to the equilibrium concentration (as t→∞); *k_KP_* is the release rate constant of the Korsmeyer–Peppas model, *n* is a diffusion exponent indicating the release mechanism: *n* = 0.5 corresponds to Fickian diffusion; *n* < 0.5 suggest an almost Fickian diffusion process; and *n* > 0.5 indicates anomalous (non-Fickian) diffusion. For the Elovich model, *Q_t_* represents the released amount of EGCG (mg/mL) at time *t*, while *α* (mg/(mL min)) is a constant related to the initial desorption rate during the burst release, and *β* (mL/mg) is the Elovich constant associated with the long-term release phase. For each of the three analyzed samples, the experimental migration data were expressed as the EGCG release fraction (*M_t_*/*M*_0_) over time, where M_0_ is the initial amount of EGCG (mg). Specifically, the mean value of *M_t_*/*M*_0_ and its variance were plotted as a function of time. To determine the parameters of the Korsmeyer–Peppas model, the data were transformed into logarithmic form by plotting ln(*M_t_*/*M*_0_) against ln(*t*), enabling the estimation of the parameter *n* and the constant *k_KH_*. Similarly, for the Elovich equation, *Q_t_* was plotted as a function of ln(*t*) thus allowing the determination of *α* and *β*. All fitting parameters are reported as mean values along with their corresponding variances.

Data fitting was performed using Origin Software 2016 (OriginLab Corporation, Northampton, MA, USA).

### 2.7. Antimicrobial and Antifungal Tests

Two plastic food bags were prepared, with the first containing a PLA_NWF sample (approximately 5 × 4 cm) previously soaked in a mixture of ethanol:H_2_O 70:30 *v*/*v*, then dried, and the second containing a PLA_NWF sample of roughly the same size but treated with a GTex extract solution and then dried (PLA_NWF_GTex). In each bag, the strawberry slices, approximately 4 mm thick, were placed directly on the cloth. The bags were then sealed and stored in light at room temperature (approximately 25 °C) for 5 days. After this period, the preservation state of the fruit was visually assessed to observe any differences.

## 3. Results and Discussion

### 3.1. Structural and Morphological Characterization of PLA-NWF and Plasma-Treated PLA-NWF

**Figure 1:**
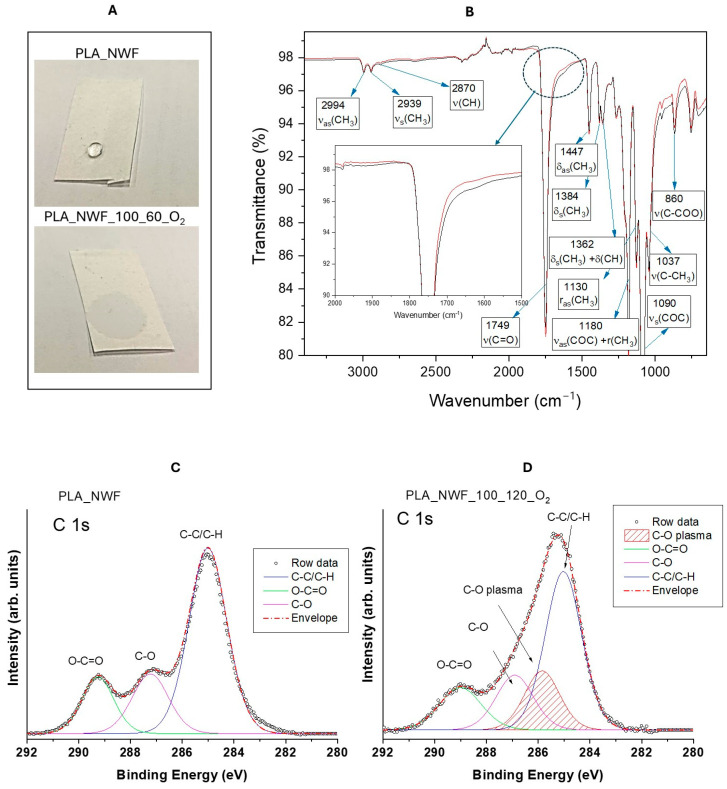
(**A**) Wettability test performed with a drop of water; (**B**) ATR-FTIR spectra of PLA_NWF sample (red curve) and PLA_NWF_100_60_O_2_ sample (black curve); (**C**,**D**) XPS spectra and relative peak deconvolution of the C1s region of PLA_NWF and PLA_NWF_100_120_O_2_ samples. ATR-FTIR spectra present the peaks, where ν, δ, and r denote stretching, bending, and rocking, with as and s representing asymmetric and symmetric, respectively.

To determine the optimal operating conditions for maximizing surface activation, we designed a protocol that enables a detailed analysis of the relationship between plasma treatment parameters and final performance. PLA-NWF samples were treated with cold plasma using three different generator power levels: 50, 75, and 100 watts. For each power level, we applied three different plasma exposure durations and oxygen or a 50/50 mixture of oxygen and argon as the plasma gas (as shown in Table S1). After the plasma treatment, the samples exhibited complete water wettability, indicating a transition from hydrophobic to hydrophilic behavior (Figure 1A).

The ATR-FTIR spectra of PLA_NWF samples displayed the diagnostic signals of PLA at 2994, 2939, 1749, 1447, 1334, 1362, 1180, 1130, 1090, 1037, and 860 cm^−1^. These peaks are attributed, as reported in Figure 1B, following the literature [44] to the stretching vibrations of –CH_3_, –C=O, and C–O–C, and –C–CH_3_ bending vibrations related to –CH_3_ and –CH groups. Notably, the carbonyl stretching band of plasma-treated specimens broadened, and a weak shoulder appeared at 1650 cm^−1^, suggesting the presence of additional surface-grafted carbonyl species (highlighted in the dotted circle and enlarged in the inset in Figure 1B) [23]. XPS analysis further supported this observation. The C1s spectra (Figure 1C) of PLA_NWF were deconvoluted into three functional components: aliphatic carbon (C–C/C-H), C–O, and O–C=O, at approximately 285, 287, and 289 eV, respectively. For the sample treated with plasma in the presence of oxygen (PLA_NWF_100_120_O_2_), a new C1s component was identified at 285.6 eV (designated as –C–O plasma), which was crucial for modeling and explaining the carbon peak profile. This component appears consistent with an oxygen-bonded, but less-polarized carbon than C=O, reasonably due to C–O–C and/or C–OH groups as already assessed in the literature [24,45,46]. Furthermore, the plasma-activated sample showed an increased quantity of C-O and O-C=O groups, while the amount of C–C/C-H decreased (Figure 1D). Concurrently, the atomic composition of oxygen increased at the expense of carbon, which decreased (refer to Figure S2A,B). These outcomes, supported by previous findings in the literature [24,27,46,47], confirmed the generation of new C-O functionalities, possibly anchored onto the surfaces of PLA-NWF, owing to cold plasma treatment.

To evaluate how plasma treatment conditions modify the surface by introducing specific functional groups, we immersed the activated samples in a buffered solution consisting of sodium acetate/acetic acid at pH 4.75 containing hexamethyl pararosaniline chloride, commonly known as crystal violet (CV). CV is a cationic dye that can establish an electrostatic interaction with polar functional groups and ionic interactions with carboxylate anion (-COO^−^). For this reason, it can be used to highlight the possible presence of these functionalities in plasma-treated samples [48]. Additionally, it can be absorbed by hydrogen bonding through hydroxyl functional groups [49].

**Figure 2:**
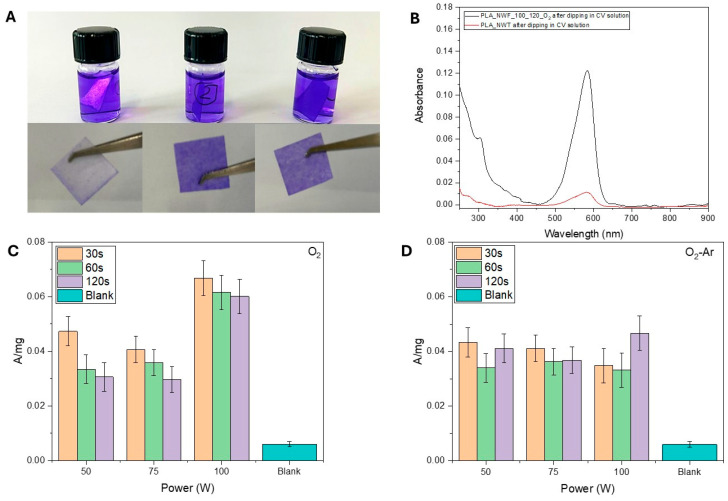
(**A**) Pictures of samples dipped in CV solution (C = 2.2 × 10^−5^ M in acetic acid/acetate buffer at pH 4.7). From left to right: PLA_NWF, PLA_NWF_100_60_O_2_, and PLA_NWF_100_60_O_2__Ar; (**B**) UV-Vis spectra of PLA_NWF and PLA_NWF_100_120_O_2_ samples after dipping in CV solution and solubilization in chloroform; normalized absorbance values (A/mg) at 592 nm of (**C**) O_2_-plasma treated samples and (**D**) O_2_-Ar-plasma treated samples (Table S1) obtained by changing the power (50, 75, 100 W) and the exposure time (30, 60, 120 s) after dipping in CV solution and solubilization in chloroform. The standard deviation was calculated based on triplicates.

The use of plasma treatment significantly alters the surface morphology and composition of PLA_NWFs, enhancing the samples’ ability to retain crystal violet (CV) dye. This results in colored surfaces that show a gradient of color intensity, which corresponds to the quantity of CV that has been immobilized (Figure 2A). The UV spectra of the solubilized samples exhibited a distinctive absorption peak for CV at 592 nm (Figure 2B). The absorbance was plotted against the experimental parameters in Figure 2C,D. The goal of this analysis is to semi-quantitatively assess the dye retention capacity based on the presence of carboxyl and/or hydroxyl functional groups, which is influenced by the plasma treatment parameters used.

As expected, all plasma-treated samples exhibited a higher dye retention capacity compared to the native sample. This confirms the presence on the surface of functional groups able to create specific interactions with CV, which is consistent with the findings from surface structural analyses conducted using ATR-FTIR and XPS.

In samples treated solely with oxygen, dye absorption increases, as the generator power increases, particularly at the 100 W setting. Conversely, the duration of plasma exposure has a relatively minor effect, with normalized absorbance typically exhibiting a downward trend. For samples treated with both oxygen and argon, the effects of power and exposure time seem less significant, as variations in absorbance values are comparable to the standard deviation of the measurements.

It is noteworthy that, as indicated in the literature [19,24], plasma treatment can lead to the formation of carbonyl compounds alongside carboxylic functionalities, a finding that was also confirmed through XPS analysis. The CV dye tends to interact preferentially with carboxylic groups [48]. Consequently, the results presented here may not entirely reflect the complete effects of the experimental parameters on the types and quantities of functionalities formed on the surface.

**Figure 3:**
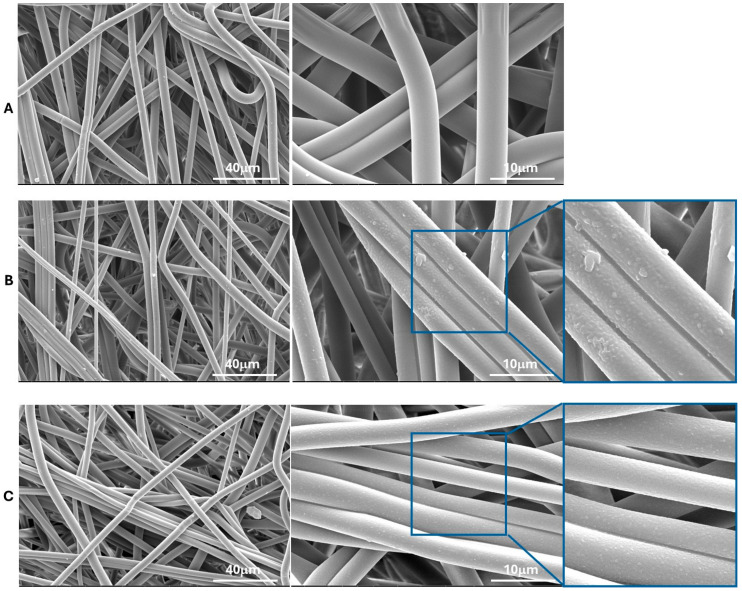
SEM micrographs at different magnifications of PLA_NWF and PLA plasma-treated samples: (**A**): PLA_NWF, (**B**): PLA_NWF_100_60_O_2_, and (**C**): PLA_NWF_100_60_O_2__Ar. On the right, a further arbitrary magnification is shown solely to highlight the observable surface modification(s).

The impact of plasma treatment on the surface morphologies of PLA-NWFs was examined using SEM, as shown in Figure 3 and Figure S3. As expected, PLA_NWF is characterized by the presence of relatively long fibers that are not perfectly uniform in diameter. These fibers are arranged in a distinctly isotropic manner and exhibit noticeable folding and twisting, as illustrated in Figure S3A. Overall, plasma treatment does not appear to result in any significant changes in the arrangement of the fibers, as demonstrated in Figure S3B,C.

Upon examining the pictures at higher magnification, the untreated, nonwoven fabric (PLA_NWF) displays a smooth and uniform surface (Figure 3A). In contrast, the plasma-treated samples exhibit significant alterations. The oxygen-treated sample is characterized by an increased surface roughness and noticeable indentations. In addition, a coating with several particles of polymeric material adhering to the fiber surfaces can be distinctly observed (Figure 3B). The sample treated with Ar and O_2_ also displayed increased roughness, along with relatively uniform surface indentations and a thinner coating (Figure 3C). This outcome is likely due to the bombardment by heavier Ar ions, which favors the formation of indentations over chemical modifications. This finding is consistent with the apparent lower degree of functionalization indicated by dye retention tests.

It has been shown that during the plasma treatment of polypropylene (PP) surfaces, a thin layer of highly functionalized polymeric material is formed. This material, occasionally soluble in water or other polar solvents, is referred to as a low-molecular-weight oxidized material (LMWOM) and can be removed from the surface [19,50]. Based on these findings, PLA_NWF and PLA_NWF_100_120_O_2_ samples were washed with ethanol and water to remove the eventually formed LMWOM. The extracts were analyzed using FTIR (Figure S4). The spectrum of the fraction extracted from PLA_NWF with ethanol shows peaks that likely correspond to plasticizers, additives, and PLA oligomers. Similar signals are also observed in the spectra of fractions extracted with water from both pristine and plasma-treated samples, although these signals are less pronounced. Alternatively, the FTIR of ethanol extract from PLA_NWF_100_120_O_2_ displays additional peaks that do not overlap with those of the PLA_NWF extracts and indicates the presence of -OH and carbonyl or carboxyl functionalities, including α-β unsaturated compounds. This suggests that the oxygen plasma treatment of PLA-NWF effectively generates a fiber coating rich in oxygenated functional groups, which is partially soluble in ethanol. This surface functionalization, which creates a layer of low-molecular-weight organic matter (LMWOM), aligns with the previous observations made for plasma-treated polypropylene (PP).

The effects of plasma treatments on bulk properties were investigated using SEC, TGA, and DSC measurements. In terms of molecular-weight evolution (Tables S2 and S3), no significant variations were observed; however, a decrease was noticed in both $\bar{M_{w}}$ (16%) and the dispersity index (Ð) of the plasma-treated samples when compared to the starting one.

### 3.2. Thermal Features of PLA-NWF and Plasma-Treated PLA-NWF

**Figure 4:**
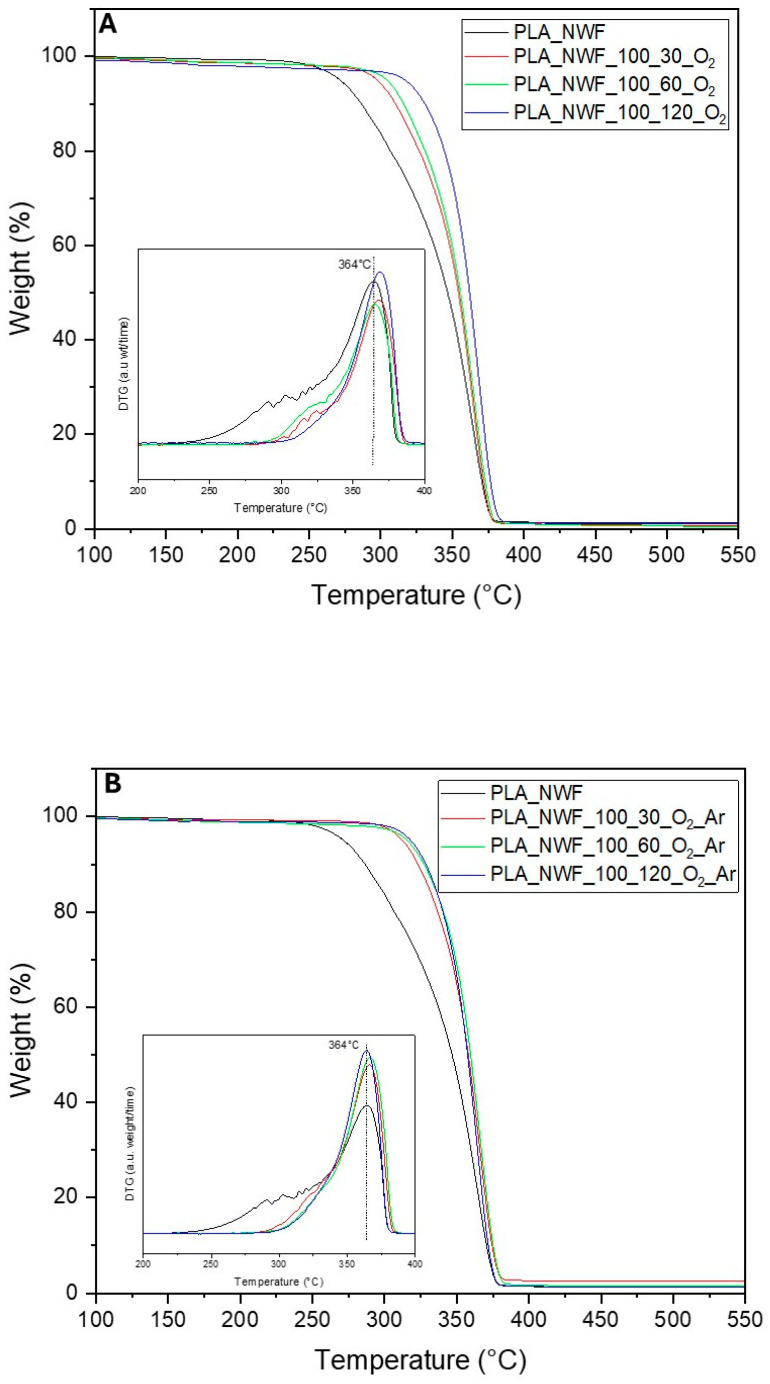
TGA analysis of pristine PLA_NWF and plasma-treated PLA_NWF: (**A**) samples treated with O_2_ and (**B**) samples treated with O_2_ and Ar.

Thermogravimetric analysis (TGA) was employed to assess the thermal stability of all produced PLA-NWF samples, treated with 100 watt plasma power for varying durations from 30 to 120 s using both O_2_ and a mixture of O_2_ and Ar gases. The thermograms of all samples displayed a similar pattern, featuring a single stage of weight loss from 30 °C to 700 °C (Figure 4). The initial degradation temperature (T_onset_) was 290 °C (calculated as the intercept from tangents to curve before and after degradation) for the control PLA_NWF sample, with a maximum degradation temperature (T_max_) of 352 °C. Following plasma treatment, an increase in thermal stability was recorded for all treated samples across varying exposure times. Specifically, T_onset_ rose to 322 °C, 330 °C, and 337 °C for the PLA_NWF_100_30_O_2_, PLA_NWF_100_60_O_2_, and PLA_NWF_100_120_O_2_ samples, respectively (Figure 4A). For samples PLA_NWF_100_30_O_2__Ar, PLA_NWF_100_60_O_2__Ar, and PLA_NWF_100_120_O_2__Ar, the T_onset_ values increased to 328 °C, 333 °C, and 333 °C (Figure 4B). In general, we observed improved stability associated with longer plasma exposure times and the use of both gases, O_2_ and Ar. Meanwhile, the maximum decomposition temperature (T_max_) remained consistent at approximately 364 °C across all plasma conditions, indicating no significant changes in bulk stability (insets in Figure 4A,B).

The observed stabilization in T_onset_ is attributed to the surface plasma treatment, in agreement with previous findings [23]. Our data further indicate that the formation of a thin layer enriched with oxidized functional groups contributes to delaying the onset of thermal degradation. Moreover, etching effects—evidenced by morphological analyses—may promote surface crosslinking phenomena, thereby providing additional stabilization. TGA analysis of the PLA_NWF_100_120_O_2_ sample after ethanol washing (Figure S5) revealed a decrease of approximately 10 °C in T_onset_ indicating that the LMWOM fraction contributes effectively to thermal stabilization. Nevertheless, the removal of this fraction does not fully restore the thermal properties of the untreated sample, suggesting that crosslinking and etching effects exert a significant influence. This interpretation is consistent with the higher T_onset_ values observed in plasma-treated samples with O_2_ and Ar, which exhibited lower degrees of functionalization but more pronounced etching.

**Figure 5:**
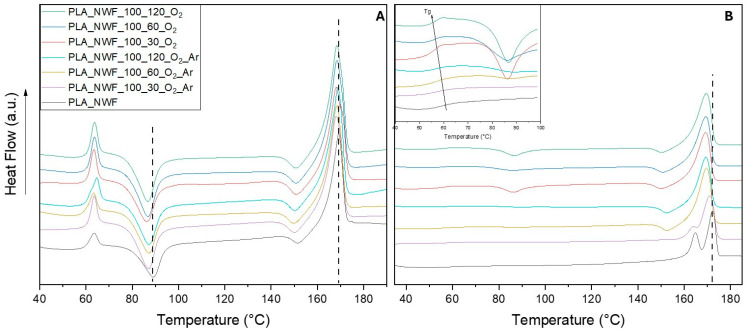
DSC thermograms of pristine PLA and plasma-treated samples: (**A**) 1st heating curves and (**B**) 2nd heating curves; inset enlargement showing the Tg and cold crystallization temperature range. The dotted lines identify the cold crystallization and melting of the PLA_NWF sample, and are shown solely to better highlight the differences with the treated samples.

DSC was conducted under standard conditions (see experimental section), with a particular focus on the first heating cycle. This cycle primarily reflects the thermal behavior of NWF after surface plasma treatment, without the influence of melting that could obscure or change the effect of plasma modification on thermal features. Three distinct thermal transitions were observed in the DSC thermogram during the first heating of PLA_NWF (Figure 5A). The first transition was the glass transition (Tg), which exhibited a relaxation endothermic peak at approximately 64 °C. This was followed by a cold crystallization process occurring around 90 °C. Lastly, a single melting endothermic peak was detected at 170 °C, along with a recrystallization exotherm observed near 151 °C. The latter peak corresponds to the rearrangement and perfection of the less-ordered α′ crystals into the thermodynamically more stable α crystals, as indicated in the literature [51,52,53].

All the plasma-treated samples displayed similar patterns characterized by the same transitions, although slightly shifted to lower temperatures. Specifically, the main cold crystallization occurred at approximately 86 °C, and the melting point was around 168 °C. This suggests that surface functionalization and indentation do not significantly impact the bulk properties of PLA-NWF, at least immediately after the plasma treatment. After cooling, the second heating analysis shows different behaviors (Figure 5B). PLA_NWF has Tg at 59.2 °C and displays two melting points at 166 °C and 172 °C, which suggests the presence of two crystalline phases (likely the α and the more disordered α’ phases) formed upon cooling and not rearranging during heating. This is supported by the significant crystallization peak observed in the cooling curve depicted in Figure S6A and the enthalpy data related to the melting and crystallization transitions presented in Table ESI4. All plasma-treated samples display a decrease in Tg values, varying between 57.9 °C and 55.6 °C, as shown in the inset of Figure 4B and Table S4. The decrease follows an almost linear trend with the duration of surface plasma exposure and the extent of modification, particularly for samples treated with oxygen. Analysis of the crystalline phases revealed that plasma-treated samples exhibited a reduction in the higher melting temperature (Tm_2_), the disappearance of the lower melting temperature, and the occurrence of crystallization due to the rearrangement of α phases at approximately 152 °C. Additionally, a pronounced delay in the crystallization process upon cooling was observed along with a remarkable reduction in both crystallization temperature (Tc) and associated enthalpies (∆Hc), as shown in Figure S6A and Table S4, for both series of samples.

These changes followed a linear trend related to the duration of plasma exposure and were especially pronounced in samples treated with O_2_. Specifically, the Tc values of the PLA_NWF_100_X_O_2__Ar samples decreased from 119.2 °C to 93.5 °C, representing a maximum percentage decrease of 38% in associated enthalpy compared to PLA_NWF. Meanwhile, the PLA_NWF_100_X_O_2_ samples exhibited Tc values around 90 °C and ∆Hc values reaching 50% of those of PLA_NWF (PLA_NWF_100_120_O_2_ sample, see Figure S6A and Table S4). A cold crystallization transition (Tcc) was also clearly noticeable at approximately 86–87 °C, even in samples subjected to shorter exposure times (PLA_NWF_100_30_O_2_, see Figure 5B and inset).

The differences in thermal behavior between pristine PLA-NWF and plasma-treated samples suggest that plasma treatment leads to the formation of less-ordered macromolecular structures and/or promotes chain arrangements into less densely packed crystalline phases. These phases are characterized by reduced crystallization (Tc) and melting (Tm) temperatures, along with lower associated enthalpy values. To further investigate this phenomenon, DSC analysis was performed on the PLA_NWF_100_120_O_2_ sample after ethanol washing (Figure S6B). The thermogram from the second heating cycle of the ethanol-washed sample closely resembled that of the untreated PLA_NWF, indicating that the LMWOM fraction is primarily responsible for the thermal changes observed in plasma-treated samples. This fraction may interact with PLA during melting, delaying subsequent crystallization and resulting in the formation of more disordered crystalline structures.

### 3.3. Coating of PLA-NWF and Plasma-Treated PLA-NWF with Green Tea Extract (GTex)

**Figure 6:**
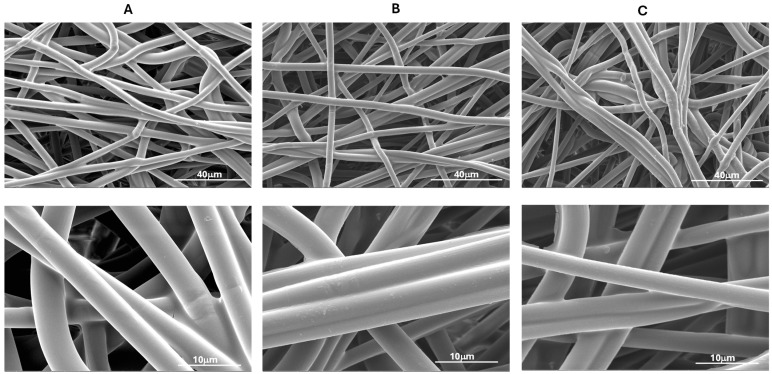
SEM images at different magnifications of samples dip-coated in GTex: (**A**) PLA_NWF_GTex; (**B**) PLA_NWF_100_60_O_2__GTex; (**C**) PLA_NWF_100_60_O_2__Ar_GTex.

Selected plasma-treated samples (PLA_NWT_100_60_O_2_ and PLA_NWT_100_60_O_2__Ar) and the untreated pristine fabric (PLA_NWF) were dip-coated with green tea extract (GTex), which is primarily composed of EGCG [54]. The concentration of EGCG in the extract was estimated to be 2.8 × 10^−2^ M using UV-vis spectroscopy (refer to the experimental section for details). Additionally, the extract was analyzed using the DPPH assay to evaluate its antioxidant effectiveness and the collected value was compared with that of pure EGCG. The EC_50_ values of GTex and EGCG are 5.4 μM and 2.1 μM, respectively. The EC_50_ value of Trolox reported in the literature is 23 μM [55]. Trolox is a water-soluble analog of vitamin E, commonly used as an antioxidant and as a reference to evaluate the antioxidant effectiveness of other antioxidants. The results suggest that GTex and EGCG have a stronger antioxidant ability than Trolox and that GTex is less effective than pure EGCG as an antioxidant. SEM images of the PLA NWF samples dip-coated with GTex reveal that the fibers are uniformly covered with a thin layer of polyphenol material (Figure 6). This coating tends to aggregate the fibers into compact and very smooth bundles. The coating effect appears homogeneous, fully adhering to the fibers while not concentrating on the spaces between them. The preservation of voids is crucial for maintaining the essential breathability properties of the nonwoven fabric. Such characteristics are particularly important for materials used in healthcare settings, like coats and face masks, which must meet specific standards for permeability and breathability [56]. Notably, the GTex coating is present and evenly distributed, even in the nonplasma-treated sample.

**Figure 7:**
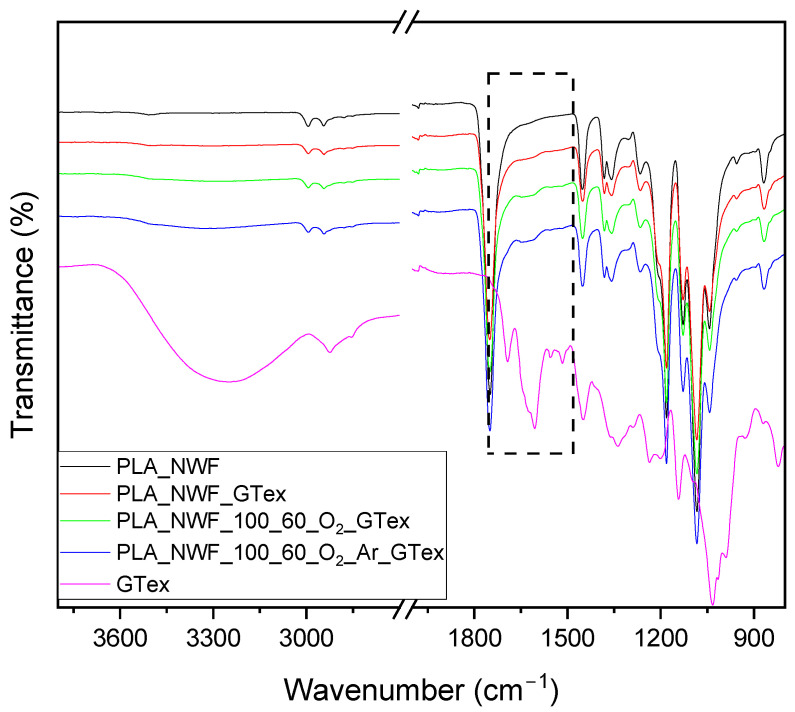
ATR-FTIR spectra of dried, extracted GTex and PLA_NWF samples dip-coated in the extract. The black dotted box emphasizes the most representative region of the spectra.

Further confirmation of the presence of EGCG in GTex and thus on the surface of fibers was achieved by ATR-FTIR (Figure 7). GTex (whose spectrum was observed after removing the mixture of solvents used for extraction) showed a wide peak centered at 3260 cm^−1^ caused by the stretching vibration of the hydroxyl (–OH) group, while the peak at 1692 cm^−1^ was due to the ester C=O. The peaks between 1609 cm^−1^ and 1450 cm^−1^ occurred due to the adsorption of aromatic C=O. The bands around 1340 cm^−1^, 1235 cm^−1^, 1200 cm^−1^, 1145 cm^−1^, and 1020 cm^−1^ can be typically correlated with the alcohol C–O, aromatic O–H, antisymmetric stretching of C–O–C, alcohol C–OH, and C–O, respectively. All signals confirm the polyphenol structure of EGCG (see Figure S7 reporting the comparison between the spectra of EGCG and GTex), definitively verifying its presence in the GTex. The spectra of samples dip-coated in the GTex displayed a shoulder in the PLA carboxyl stretching and a small additional peak around 1630 cm^−1^ (as shown in the black box with the dotted line in Figure 7). This peak, which is indicative of GTex, increases from PLA_NWF_GTex to PLA_NWF_100_60_O_2__GTex. This suggests that plasma-treated samples have a greater capacity to absorb the GTex.

TGA analysis of dip-coated samples, carried out under nitrogen, allowed for determining their thermal stability and the content of GTex present on their surfaces (Figure S7A). GTex shows a multiple-step degradation pattern already discussed in the literature [54]: a first weight loss from 50 °C to 180 °C due to volatiles and absorbed water, followed by the non-oxidative degradation of polyphenols, which started at about 200 °C with a mass loss of about 15% and proceeded with a second step from 180 to 280–300 °C related to the main degradation of catechin derivatives. All PLA samples treated with GTex exhibit a degradative pathway in two steps. Compared with the untreated sample, they show a first onset at a lower temperature (about 200 °C) due to the loss of the volatile or less stable component of the extract (see blue arrow in both Figure S8A,B) and also a second step (see magenta arrow in Figure S8A), presumably attributable to PLA starting degradation, occurring at a higher temperature than the pristine fabric (301 °C vs. 290 °C). Instead, a lower T_inf_ of 10 °C was observed for GTex-treated PLA (dotted line in Figure S8B), suggesting a weak decrease in the thermal stability of PLA-NWF aligning with the lower stability of GTex covering the fibers. The amount of residue allowed us to estimate the GTex content absorbed by the various samples (Table 1). As expected in the case of samples previously activated by plasma treatment, the content of absorbed GTex was notably higher than that of untreated PLA-NWF. Finally, to estimate the amount of EGCG adsorbed by each sample through GTex-covering, the dipped fabrics were dissolved in chloroform, and solutions were analyzed by UV-vis spectroscopy (Figure S9). Samples show a signal at 278 nm that can be associated with the characteristic absorption of EGCG (at 273 nm) whose spectrum in ethanol is shown in Figure S9 as the inset. Taking into account the relative weights of the NWF samples and the value of the absorption maximum, the weight content of EGCG absorbed on each sample was estimated, finding a trend consistent with the data extrapolated from TGA analyses (Table 1). Even if this method allows for a rough quantification of EGCG uptake, it confirms the effectiveness of plasma treatment on the coating yield of the PLA-NWF samples: the highest deposition of the extract was found in the case of plasma-treated samples (particularly for the oxygen-treated one) confirming the superior ability of this sample to anchor both dye molecules and polyphenols on its surface.

### 3.4. Migration Tests

To investigate the impact of surface modifications on the release profile of EGCG from PLA_NWF samples, deionized water was selected as the release medium, given EGCG’s high water solubility (521.7 g/L) [57]. After drying and characterization, EGCG-functionalized PLA_NWF samples were immersed in water, and their EGCG release over time was monitored via UV-vis spectroscopy.

**Figure 8:**
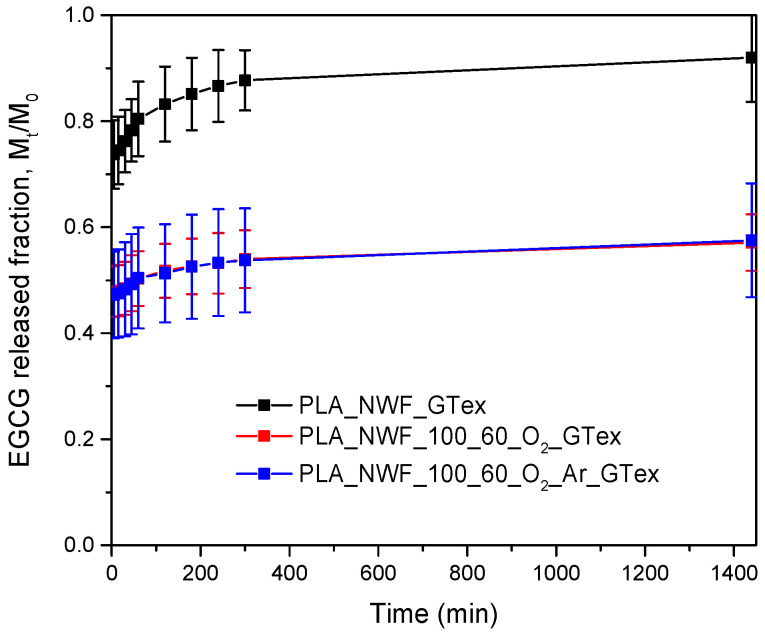
Release profile in water of EGCG from PLA_NWF_GTex, PLA_NWF_100_60_O_2__GTex, and PLA_NWF _100_60_O_2__Ar_GTex.

Data analysis showed that the experimentally measured EGCG released fraction (*M_t_*/*M*_0_) varied significantly between plasma-treated and untreated samples (Figure 8). After 24 h, the untreated PLA_NWF_GTex sample exhibited a released fraction of approximately 0.9, whereas the two plasma-treated samples, PLA_NWF_O_2__GTex and PLA_NWF_O_2__Ar_GTex, showed *M_t_*/*M*_0_ values around 0.6. These results suggest that EGCG release is substantially higher in the untreated PLA_NWF, while both plasma-treated samples, whether treated with O_2_ or an O_2_-Ar mixture, exhibited similar behavior.

Two main factors can account for such a phenomenon. First, the interaction between EGCG and the functional groups introduced on the PLA_NWF surface during plasma treatment may affect compound release. Second, the newly developed rougher fiber morphology—evidenced by SEM analyses—could trap EGCG within the micro-roughness, slowing the release kinetics. This aligns with the previous studies on other substrates [58].

The rapid initial release phase is another notable aspect. A significant portion of the surface-deposited EGCG was released immediately after the immersion of the samples in water. This sharp increase, over half of the total released compound desorbed within the first 5 min, is characteristic of burst release [59,60,61,62]. This phenomenon results from weakly adhered molecules dissolving immediately upon contact with water. However, after approximately 10 h, the desorption process slowed significantly, eventually stabilizing as the system approached equilibrium.

A preliminary review of the collected trends indicates that the observed behavior does not adhere to a zero-order kinetic model, as the release rate varies over time. Additionally, a first-order model cannot explain the release because, in such cases, the amount released should be directly proportional to the number of molecules incorporated within the matrix at each time point, which is not evident in the experimental data. Furthermore, the release of EGCG does not follow a pure Fickian diffusion mechanism, as the amount released is not proportional to the square root of time [58,63]. These findings suggest that additional factors may be involved, such as specific interactions with the material’s fibers.

To better describe the release kinetics, two models were applied, accounting for both diffusional factors and the surface dissolution of molecules, which more accurately represent our situation. The Korsmeyer–Peppas model, widely used for analyzing polyphenol release from polymeric systems [20,64,65], describes the release kinetics through a mathematical expression, where the parameter *n* provides insights into the diffusion mechanism and *k_KP_* represents the kinetic release constant. This model accounts for a confined diffusion effect, in which adsorbed molecules move along the fiber surface before release [66]. In contrast, the Elovich model is commonly employed to describe adsorption and desorption processes, characterizing a release mechanism mediated by surface dissolution [67,68].

This model relies on two key parameters: α, which is related to the short-term release rate and describes the burst release (i.e., the rapid initial release of EGCG), and β, associated with long-term release, indicating the sustainability of the release over time. Higher α values suggest a more pronounced initial burst release, while higher β values indicate a more prolonged and controlled release.

**Figure 9:**
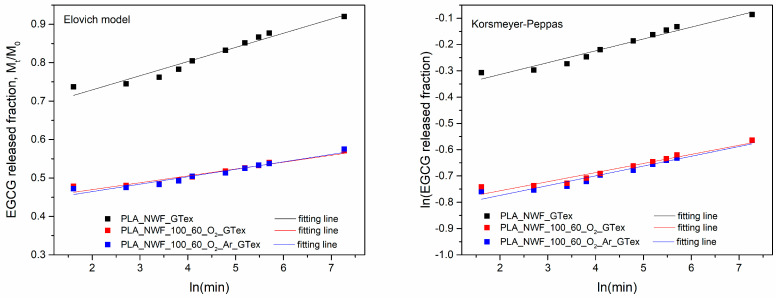
Elovich and Korsmeyer–Peppas fittings of EGCG water release from PLA_NWF_GTex, PLA_NWF_O_2__GTex, and PLA_NWF_O_2__Ar_GTex.

The fitting results (Figure 9) show that the three samples’ release behavior fits well with the Elovich model and the Korsmeyer–Peppas model, with R^2^ values exceeding 0.9 (Table 2).

Regarding the Korsmeyer–Peppas model, the *n* parameter is similar for all samples and remains below 0.5, indicating a quasi-Fickian release behavior. On the other hand, the release constant is identical across samples. This model primarily fits the data following the burst release phase and suggests that all samples exhibit similar release kinetics.

In the case of the Elovich model, *α* values are higher in plasma-treated samples than in untreated ones. This effect is likely due to the initial dissolution of non-bound molecules deposited in more significant amounts in treated samples. This result confirms the adequacy of the model in describing the experimental data. The *β* parameter is similar across all samples, although the PLA_NWF_100_60_O_2__GTex sample exhibits the lowest value, suggesting a slower desorption rate in the second phase. This phenomenon may be related to an enhanced interaction between EGCG and the treated fibers [69]. Moreover, it cannot be excluded that this effect may also be due to the migration of functionalized LMWOM formed on the surface, which can interact with EGCG and migrate more slowly than the free molecule.

### 3.5. Thermal–Oxidative Study and Evaluation of the Antimicrobial Behaviour of PLA-NWF and PLA-NWF Coated with GTex

**Figure 10:**
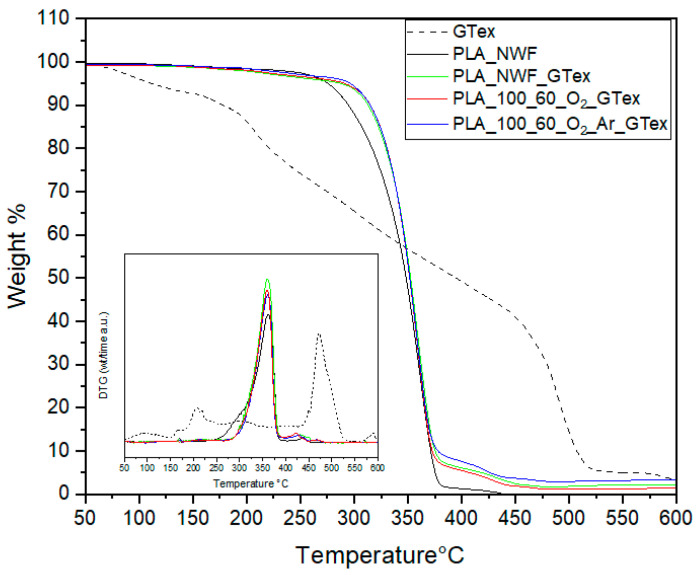
TGA-under-air results of GTex, PLA_NWF, PLA_NWF_GTex, PLA_NWF_100_60_O_2__Ar_GTex, and PLA_NWF_100_60_O_2__GTex; inset: DGT curves.

EGCG is widely recognized for its potent antioxidant properties, which can enhance the durability of biopolymer-based materials, facilitating the development of fully biobased plastic formulations. To evaluate the effectiveness of this natural additive as a stabilizer against thermo-oxidative degradation, TGA was performed in the presence of air to determine the onset degradation temperature (T_onset_) of PLA-NWF treated with GTex under oxidative conditions (see Figure 10). Although the T_onset_ of GTex was relatively low (the first step was at about 115 °C), it demonstrated a significant stabilizing effect on PLA-NWF. The T_onset_ increased from 247 °C for PLA_NWF to 288 °C for PLA_NWF_GTex. Furthermore, it rose further to 291 °C and 296 °C for the PLA_NWF_100_60_O_2__Ar_GTex and PLA_NWF_100_60_O_2__GTex samples, respectively. These findings indicate that EGCG, absorbed onto the surface of PLA-NWF, effectively mitigates thermo-oxidative degradation, with the stabilizing effect appearing to be proportional to the amount of EGCG present.

**Figure 11:**
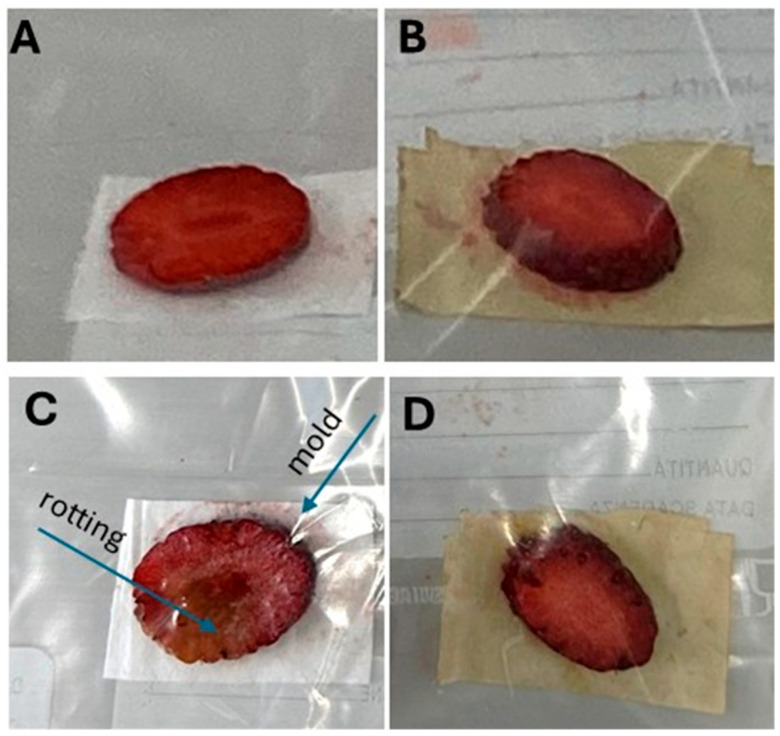
The application of untreated PLA_NWF and PLA_NWF treated with GTex on packaged strawberry slices; (**A**,**B**) strawberry slices packed with PLA_NWF and PLA_NWF_GTex; (**C**,**D**) strawberry slices packed with PLA_NWF and PLA_NWF_GTex after 5 days.

To preliminarily assess the antimicrobial and antifungal properties of PLA-NWF treated with GTex, strawberry slices were placed inside a plastic bag [70]. In the first experimental set (Figure 11A), one piece of strawberry was placed in direct contact with a sample of PLA_NWF treated with a 70/30 ethanol/water mixture and then dried. The same setup was repeated with a second sample consisting of PLA_NWF treated with GTex (Figure 11B). After five days of storage at room temperature, a visual inspection revealed that the strawberry slices placed on the untreated PLA_NWF exhibited clear signs of degradation, including rotting and mold growth (Figure 11C). In contrast, the samples in contact with PLA_NWF treated with GTex remained almost entirely intact, showing little to no signs of significant organic deterioration (Figure 11D). Although totally preliminary and requiring further confirmation, these results suggest that EGCG, the active compound in green tea, transferred to PLA-NWF, can effectively inhibit biofilm and mold formation.

## 4. Conclusions

This study explores the surface modification of polylactic acid, nonwoven fabrics (PLA-NWF) using cold plasma treatment with oxygen and an oxygen/argon gas mixture. Both treatments successfully introduced oxygenated functional groups onto the fabric surfaces, improving wettability and enhancing interactions with dyes and polyphenols. Oxygen plasma treatment alone allows tunable modification based on generator power and exposure time, whereas the addition of argon has a marginal effect on chemical functionalization but increases surface roughness due to greater fiber indentation.

Extraction analysis revealed that plasma treatment forms a highly functionalized, oxygen-rich surface layer on the PLA-NWF surface. This layer is partially soluble in ethanol and influences the bulk properties of the material. Specifically, it delays thermal degradation and modifies the thermal transitions (melting and crystallization) of PLA after the first melting cycle. Importantly, this layer plays a key role in enhancing the fabric’s ability to absorb active molecules, such as those in green tea extract (GTex), with particular reference to epigallocatechin gallate (EGCG), which was observed to uniformly coat the fiber surfaces.

Migration tests showed that plasma treatment significantly reduces EGCG release compared to untreated fabrics. This effect is attributed to stronger interactions between EGCG and the plasma-modified surface, aided by increased surface roughness that hinders diffusion. Although the overall release kinetics were similar, fabrics treated with the O_2_–plasma exhibited a slower desorption phase after the initial burst release.

Thermogravimetric analysis demonstrated that PLA fabrics loaded with EGCG possess improved thermo-oxidative stability, thanks to the antioxidant activity of the extract. The degree of protection correlated with the amount of retained extract. Preliminary antimicrobial and antifungal tests suggest that GTex-treated fabrics can extend food shelf life.

These results highlight the potential of cold plasma surface treatments to functionalize bioplastic nonwoven fabrics for enhanced absorption and the controlled release of natural bioactive molecules. Such materials hold promise for applications in sustainable packaging, cosmetics, and biomedical devices. Further studies will examine their antioxidant, UV-blocking, and antibacterial properties.

## Figures and Tables

**Table 1:** Content of absorbed GTex and EGCG on dip-coated samples.

Sample	wt % of GTex ^1^	wt % of EGCG ^2^
PLA_NWF_GTex	4.4	3.1
PLA_NWF_100_60_O_2__GTex	10.5	7.4
PLA_NWF_100_60_O_2__Ar_GTex	9.8	5.8

^1^ Determined from the percentage of residues (in TGA analysis); ^2^ determined from UV-Vis absorbance values of EGCG in the solubilized dip-coated PLA_NWF samples. Calculation was performed by considering A_275_–A_900_ to eliminate any effect owing the differences in the baseline. The resulting data were used to calculate the concentration of EGCG.

**Table 2:** Fitting parameters of the Elovich and Korsmeyer–Peppas equations extracted through the nonlinear fitting of experimental data points.

	Elovich			Korsmeyer-Peppas		
Sample	*α* ^a^ × 10^5^ (mg mL^−1^ min^−1^)	*β* ^b^ (mL mg^−1^)	R^2 c^	*n* ^d^	*k_KP_* ^e^ (min^−n^)	R^2 c^
PLA_NWF_GTex	0.40 ± 0.01	919 ± 73	0.95	0.045 ± 0.001	0.726 ± 0.007	0.96
PLA_NWF_O_2__GTex	660 ± 11	863 ± 80	0.94	0.036 ± 0.003	0.767 ± 0.020	0.94
PLA_NWF_O_2__Ar_GTex	95 ± 2	1021 ± 91	0.94	0.037 ± 0.004	0.745 ± 0.022	0.95

^a^ Elovich: initial release rate of the active compound. ^b^ Elovich: Elovich constant associated with the long-term release phase. ^c^ Coefficient of determination of the model. ^d^ Korsmeyer–Peppas model: diffusional exponent. ^e^ Korsmeyer–Peppas model: release rate constant.

## Data Availability

The original contributions presented in this study are included in the article and/or in Supplementary Materials.

## Supplementary Materials

The following supporting information can be downloaded at: https://www.mdpi.com/article/10.3390/polym17111482/s1, Figure S1: Calibration curve for determining the concentration of EGCG in the GTex obtained by measuring the absorbance of ethanol:water (3:1) solutions at different concentrations of pure, standard EGCG. Specifically, the measurements were performed on samples obtained by diluting in an ethanol:water (3:1) solvent mixture the ethanol stock solution of ECGG. Inset UV-vis spectrum of diluted GTex (10μL of GTex in 5 mL of ethanol:water (3:1) solutions), R^2^ value of fitting = 0.99982; Figure S2: (A) Content of the different carbon-bearing species and (B) Shape of the O 1s XPS peak before and after plasma treatment; Figure S3: SEM images of A: PLA_NWF, B: PLA_NWF_100_60_15_O_2_, and C: PLA_NWF_100_60_15_O_2__Ar; Figure S4: FTIR of water and ethanol extracted fractions from pristine and cold plasma (oxygen)-treated samples; -C=O and C=C stretching regions are shown in the green circle; Figure S5: TGA (black) and DTG (red) patterns of PLA_NWF_100_120_O_2_ sample before (straight line) and after (dashed line) washing with EtOH; Figure S6: A: DSC thermograms (cooling curves) of pristine PLA_NWF and plasma-treated samples; B: cooling and 2nd heating curves of PLA_NWF and PLA_NWF_100_120_O_2_ samples after washing with EtOH; Figure S7: FTIR-ATR spectra of GTex (black curve) and EGCG standard (red curve); Figure S8: A: TGA and B: DTG curves of GTex and PLA_NWF samples dip-coated with GTex. Analysis carried out under nitrogen; Figure S9: UV-Vis spectra of PLA_NWF_GTex, PLA_NWF_100_60_O_2__GTex, and PLA_NWF_100_60_O_2__Ar_GTex samples solubilized in chloroform; inset UV-Vis spectrum of pure EGCG solubilized in EtOH (C = 2.5 × 10^−3^). Table S1: Plasma treatment conditions of PLA_NWF; Table S2: Molecular weights ($\bar{M_{n}}$, $\bar{M_{w}}$) and dispersity index (Ð) of PLA_NWF and samples treated with plasma in the presence of oxygen; Table S3: Molecular weights ($\bar{M_{n}}$, $\bar{M_{w}}$) and dispersity index (Ð) of PLA_NWF and samples treated with plasma in the presence of oxygen and argon; Table S4: DSC data from cooling and 2nd heating curves of PLA_NWF and plasma-treated samples.
