# Supplementary material for: Enhancing the Performance of PLA Nonwoven Fabrics Through Plasma Treatments for Superior Active-Molecule Retention
Source: Polymers (Basel). 2025 May 27;17(11):1482. doi: 10.3390/polym17111482 (PMC12157710; doi:10.3390/polym17111482)
Supplement: Supplementary file 1 [file polymers-17-01482-s001.zip › polymers-3622955-supplementary.pdf]

Electronic Supporting Information

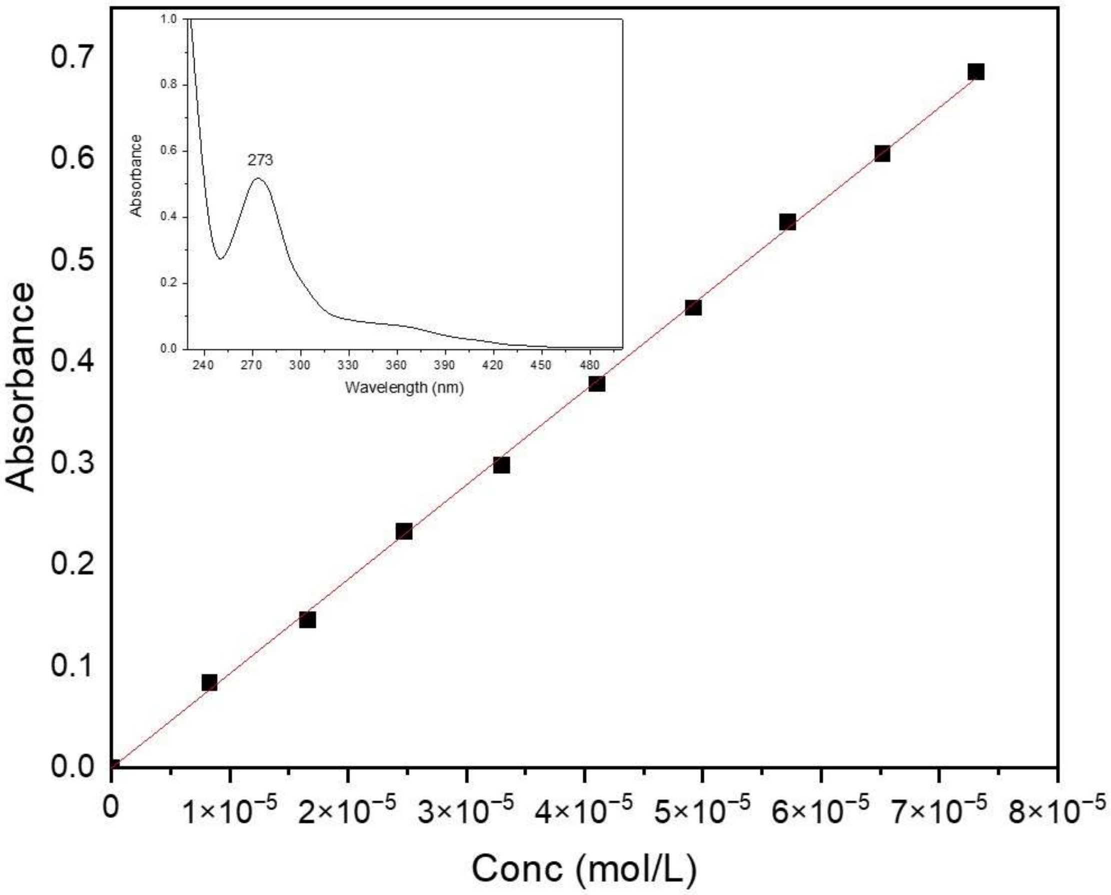

Figure S1: Calibration curve for determining the concentration of EGCG in the GTex obtained by measuring the absorbance of ethanol:water 3:1 solutions at different concentrations of the pure, standard EGCG. Specifically, the measurements were done on samples obtained by diluting in ethanol:water 3:1 solvent mixture the ethanol stock solution of ECGG. Inset UV-vis spectrum of diluted GTex (10μL of GTex in 5mL of ethanol:water 3:1 solutions). R^2 value of fitting = 0.99982.

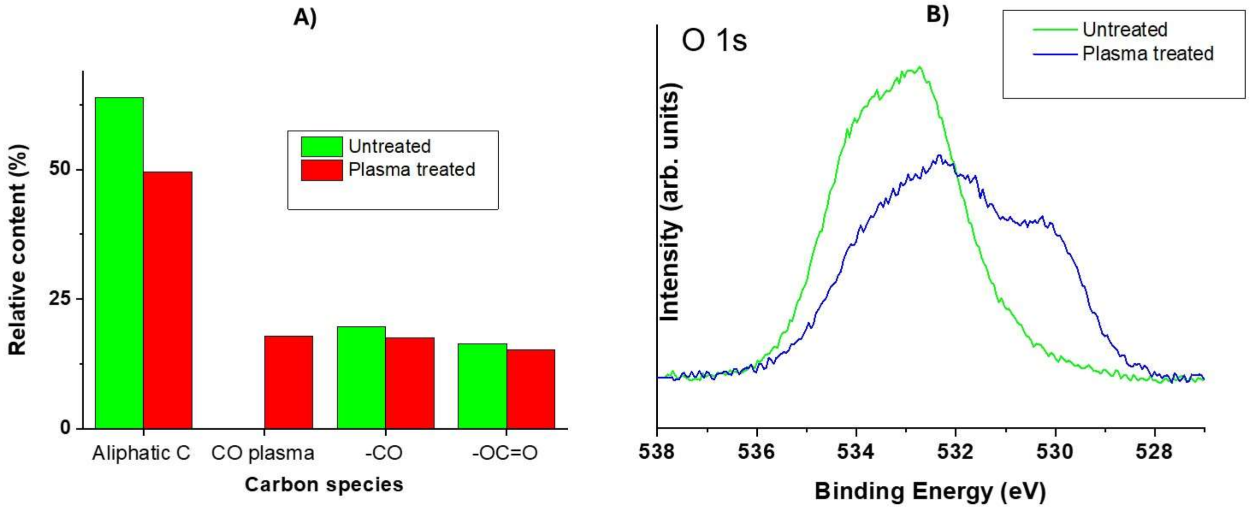

Figure S2: A) Content of the different carbon-bearing species and B) Shape of the O 1s XPS peak before and after plasma treatment.

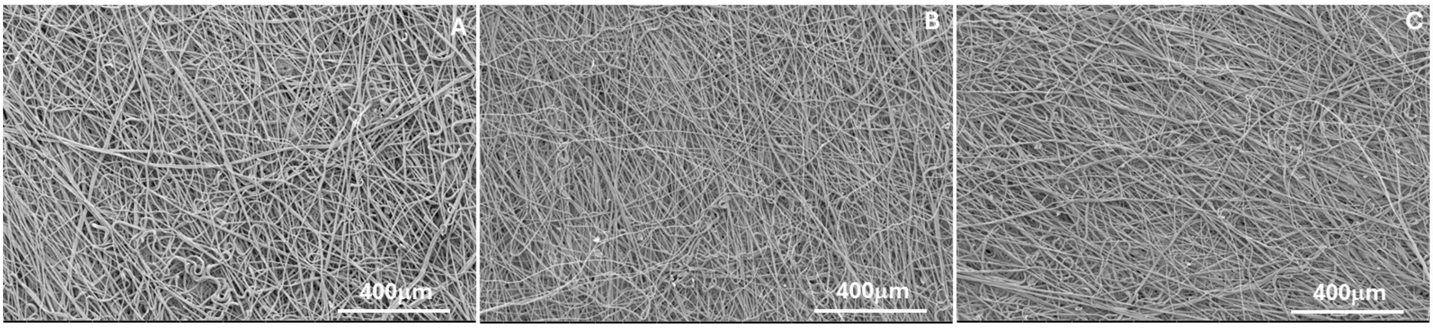

Figure S3: SEM images of A: PLA_NWF, B: PLA_NWF_100_60_15_O_2 and C: PLA_NWF_100_60_15_O_2_Ar

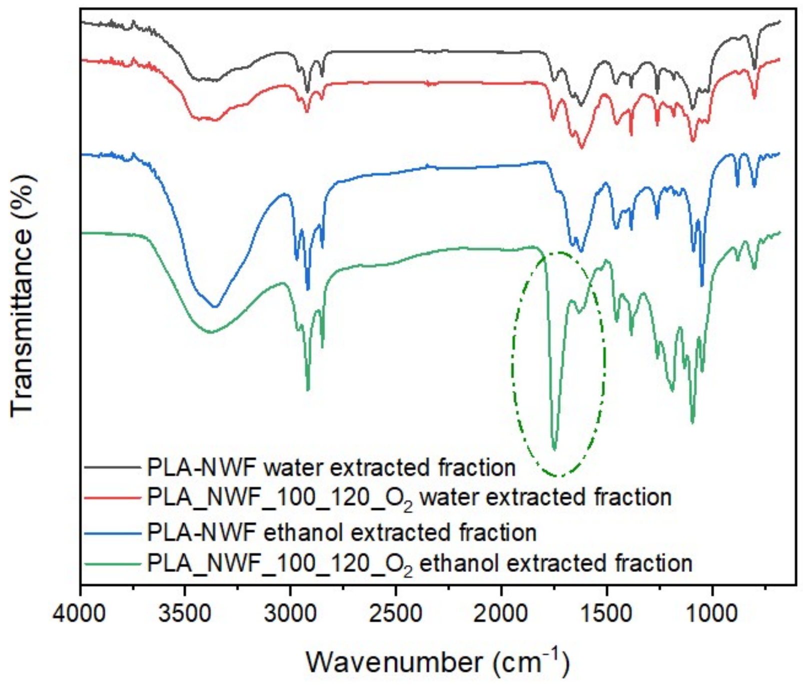

Figure S4: FTIR of water and ethanol extracted fractions from pristine and cold plasma (oxygen) treated samples; -C=O and C=C stretching regions are evidenced in the green circle.

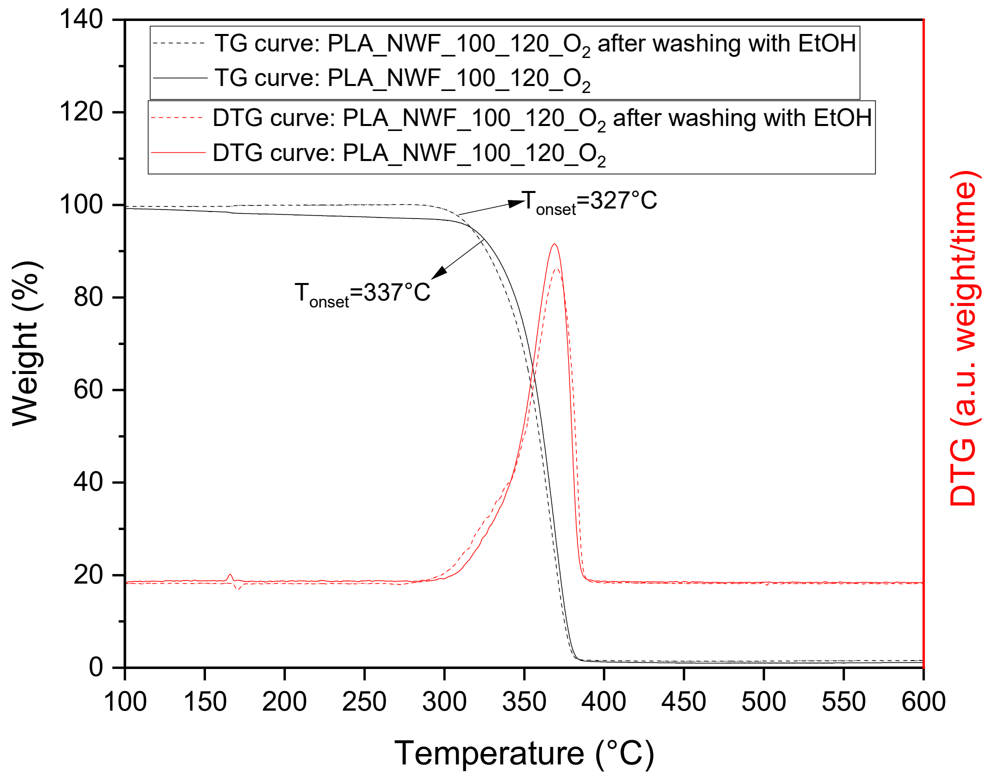

Figure S5: TGA (black) and DTG (red) patterns of sample PLA_NWF_100_120_O_2 before (straight line) and after (dashed line) washing with EtOH.

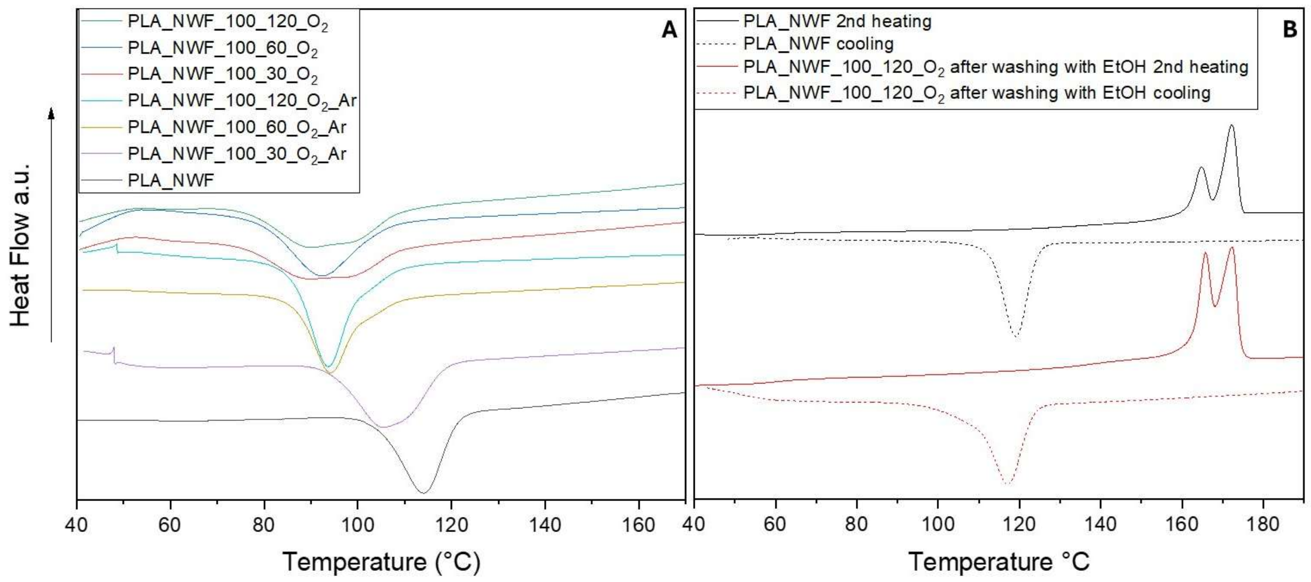

Figure S6. A: DSC thermograms (cooling curves) of pristine PLA_NWF and plasma-treated samples; B: colling and 2nd heating curves of PLA_NWF and PLA_NWF_100_120_O_2 sample after washing with EtOH

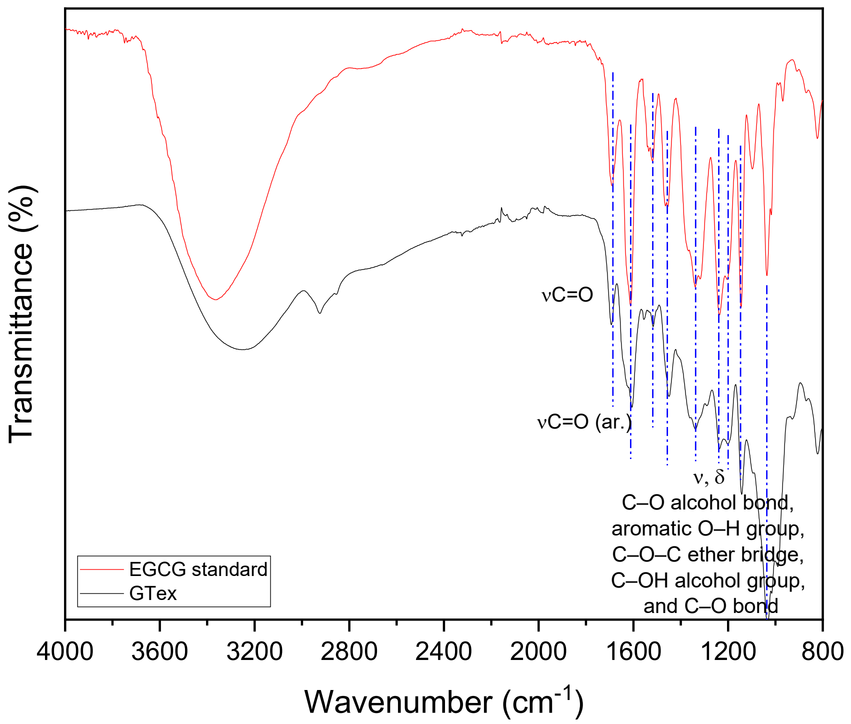

Figure S7: FTIR-ATR spectra of GTex (black curve) and EGCG standard (red curve)

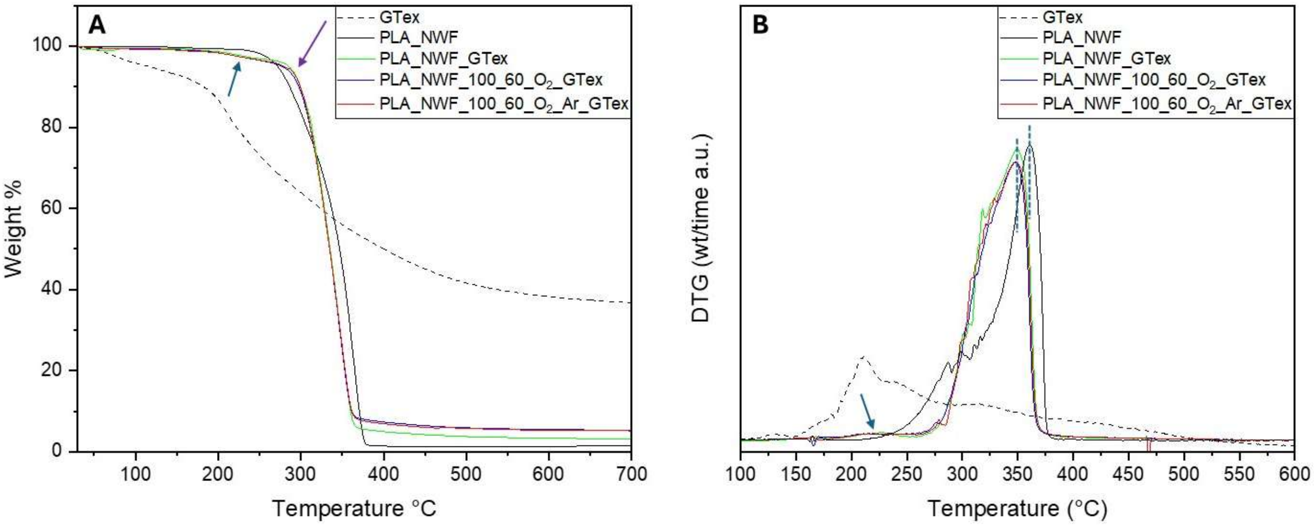

Figure S8. A: TGA and B: DTG curves of GTex and PLA_NWF samples dip-coated with GTex. Analysis carried out under nitrogen.

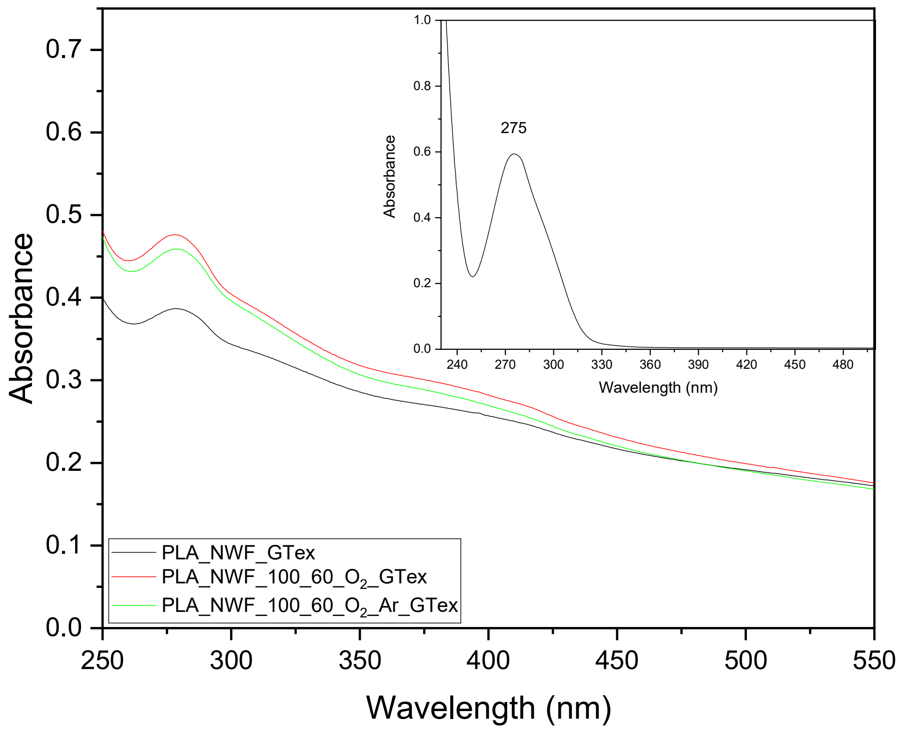

Figure S9: UV-Vis spectra of samples PLA_NWF_GTex; PLA_NWF_100_60_O_2_GTex; PLA_NWF_100_60_O_2_Ar_GTex solubilized in chloroform; inset UV-Vis spectrum of pure EGCG solubilized in EtOH (C=2.5*10^$^{-3}$)

Table S1. Plasma treatment conditions* of PLA_NWF

Sample name	Plasma Generator Power (W)	Time (s)	Oxygen flux (sccm)	Argon flux (sccm)
PLA_NWF (Blank)	-	-	-	-
PLA_NWF_50_30_O_2	50	30	15	-
PLA_NWF_50_60_O_2	50	60	15	-
PLA_NWF_50_120_O_2	50	120	15	-
PLA_NWF_75_30_O_2	75	30	15	-
PLA_NWF_75_60_O_2	75	60	15	-
PLA_NWF_75_120_O_2	75	120	15	-
PLA_NWF_100_30_O_2	100	30	15	-
PLA_NWF_100_60_O_2	100	60	15	-
PLA_NWF_100_120_O_2	100	120	15	-
PLA_NWF_50_30_O_2_Ar	50	30	15	15
PLA_NWF_50_60_O_2_Ar	50	60	15	15
PLA_NWF_50_120_O_2_Ar	50	120	15	15
PLA_NWF_75_30_O_2_Ar	75	30	15	15
PLA_NWF_75_60_O_2_Ar	75	60	15	15
PLA_NWF_75_120_O_2_Ar	75	120	15	15
PLA_NWF_100_30_O_2_Ar	100	30	15	15
PLA_NWF_100_60_O_2_Ar	100	60	15	15
PLA_NWF_100_120_O_2_Ar	100	120	15	15

* Samples were treated on both sides.

Table S2. Molecular weights ($\overline{M_n}$, $\overline{M_w}$) and dispersity index (Đ) of the PLA_NWF and samples treated with plasma in the presence of oxygen

Sample	$\overline{M_n}$(g/mol)	$\overline{M_w}$ (g/mol)	Đ
PLA_NWF	65.800	124.200	1.9
PLA_NWF_50_30_O_2	65.400	111.400	1.7
PLA_NWF_50_60_O_2	65.200	111.500	1.7
PLA_NWF_50_120_O_2	62.600	106.600	1.7
PLA_NWF_75_30_O_2	61.200	109.800	1.8
PLA_NWF_75_60_O_2	64.800	109.900	1.7
PLA_NWF_75_120_O_2	65.600	110.700	1.7
PLA_NWF_100_30_O_2	64.000	111.800	1.7
PLA_NWF_100_60_O_2	66.600	107.800	1.6
PLA_NWF_100_120_O_2	60.500	105.900	1.7

Table S3. Molecular weights ($\overline{M_n}$, $\overline{M_w}$) and dispersity index (Đ) of the PLA_NWF and samples treated with plasma in the presence of oxygen and argon

Sample	$\overline{M_n}$(g/mol)	$\overline{M_w}$ (g/mol)	PDI
PLA_NWF	65.800	124.200	1.9
PLA_NWF_50_30_O_2_Ar	62.000	107.000	1.7
PLA_NWF_50_60_O_2_Ar	63.000	108.600	1.7
PLA_NWF_50_120_O_2_Ar	62.400	106.300	1.7
PLA_NWF_75_30_O_2_Ar	62.400	107.300	1.7
PLA_NWF_75_60_O_2_Ar	65.700	109.100	1.7
PLA_NWF_75_120_O_2_Ar	61.000	108.900	1.8
PLA_NWF_100_30_O_2_Ar	63.200	108.800	1.7
PLA_NWF_100_60_O_2_Ar	62.500	107.400	1.7
PLA_NWF_100_120_O_2_Ar	61.900	105.600	1.7

Table S4: DSC data from cooling and 2nd heating curves of PLA_NWF and plasma treated samples

Sample	T_g* (°C)	T_{m1}* (°C)	T_{m2}* (°C)	ΔH_{m1}* (J/g)	ΔH_c§ (J/g)	T_c§ (°C)	ΔH_{cc}* (J/g)	T_{cc1}* (°C)	T_{cc2}* (°C)
PLA_NWF	59.2	166.0	172.0	57.1	-51.6	119.1	-	-	-
PLA NWF_100_30_O_2_Ar	57.9	164.0	171.0	50.6	-47.6	105.8	-	-	-
PLA NWF_100_60_O_2_Ar	56.8	-	169.6	39.8	-32.2	94.2	-	-	152.0
PLA NWF_100_120_O_2_Ar	57.2	-	169.4	51.8	-43.3	93.5	-	-	152.0
PLA NWF_100_30_O_2	56.3	-	169.3	38.6	-30.3	89.8	9.5	86.6	149.9
PLA NWF_100_60_O_2	55.8	-	169.3	47.0	-39.8	92.6	6.6	86.0	150.9
PLA NWF_100_120_O_2	55.6	-	169.6	49.4	-24.2	90.1	9.9	86.6	150.3

*: Tg, melting ($m_1$and m_2) and cold crystallization (cc) data collected from 2nd heating curves (Figure 2B); ΔH_{cc} is related to T_{cc1}

§: crystallization (c) data collected from cooling curves (Figure ESI6A)
